# Queensland Genomics: an adaptive approach for integrating genomics into a public healthcare system

**DOI:** 10.1038/s41525-021-00234-4

**Published:** 2021-08-18

**Authors:** Miranda E. Vidgen, Dayna Williamson, Katrina Cutler, Claire McCafferty, Robyn L. Ward, Keith McNeil, Nicola Waddell, David Bunker

**Affiliations:** 1 QIMR Berghofer Medical Research Institute, Herston, QLD Australia; 2 Queensland Genomics, Queensland Health, Herston, QLD Australia; 3 Faculty of Health, Queensland University of Technology, Kelvin Grove, QLD Australia; 4 Brisbane Diamantina Health Partners, The University of Queensland, Herston, QLD Australia; 5 Faculty of Medicine, The University of Queensland, Herston, QLD Australia; 6 Faculty of Medicine and Health, University of Sydney, Camperdown, NSW Australia; 7 Offices of Chief Medical Officer and Chief Clinical Information Officer, Queensland Health, Brisbane, QLD Australia

**Keywords:** Genetics research, Genetic services, Translational research, Molecular medicine, Health policy

## Abstract

The establishment of genomics in health care systems has been occurring for the past decade. It is recognised that implementing genomics within a health service is challenging without a system-wide approach. Globally, as clinical genomics implementation programs have matured there is a growing body of information around program design and outcomes. Program structures vary depending on local ecosystems including the health system, politics and funding availability, however, lessons from other programs are important to the design of programs in different jurisdictions. Here we describe an adaptive approach to the implementation of genomics into a publicly funded health care system servicing a population of 5.1 million people. The adaptive approach enabled flexibility to facilitate substantial changes during the program in response to learnings and external factors. We report the benefits and challenges experienced by the program, particularly in relation to the engagement of people and services, and the design of both individual projects and the program as a whole.

## Introduction

Information from genome sequencing can be used to inform patient care and is at a point where its mainstream use in various medical specialities is possible^1,2^. The ability to translate genomics into clinical practice is complex and requires tailored system-wide changes to health organisations^3^, and may require a re-assessment of how genomic services and value in healthcare are evaluated.

To realise the potential of genomics in healthcare, the challenges it poses need to be addressed systematically. Challenges include: increasing patients and community awareness of genomics and familial implications; training a clinical workforce to apply genomics in clinical practice; establishing infrastructure for genomic testing, analysis and information systems for genomic data management, sharing, integration and long-term data usage; providing evidence on the value of genomics to the healthcare system and patient care; and addressing concerns about ethical, legal and social issues raised by the application of genomic medicine^4–7^. The level of interrelated system-wide changes required for the incorporation of genomics into routine care likely results in fragmented and costly adoption of genomic services^8^.

There has been a global investment in the implementation of genomics into healthcare systems, with multiple countries or jurisdictions establishing programs^9–13^. Each program has set out to address different issues related to the uptake and management of genomics in clinical settings, fundamental research, and translation of genomic research. These approaches have varied, reflecting local requirements, context and end goals.

Public healthcare in Australia is a joint responsibility of the Federal Government and the seven states and territories. Each state manages its own public health services. In the last five years investment in genomics has occurred through a single national research program (Australian Genomics)^9^ as well as individual state/territory-based programs^3,13^. These programs have had significant success in creating political and health systems support for clinical genomics in all Australian public health systems. However, the Australian context of a federated healthcare system has resulted in implementation at varying levels of maturity, reflecting each jurisdiction’s healthcare system and research environment.

This paper describes the genomics program in one state, Queensland, where health services are governed and delivered through 16 Hospital and Health Services as statutory bodies^14,15^ utilising a budget of $AU15.6 billion (2019–20). This publicly funded system provides healthcare for a population of 5.1 million people over a geographical area seven times the size of the United Kingdom.

## Program structure and processes

### Program at the time of establishment

**Fig. 1:**
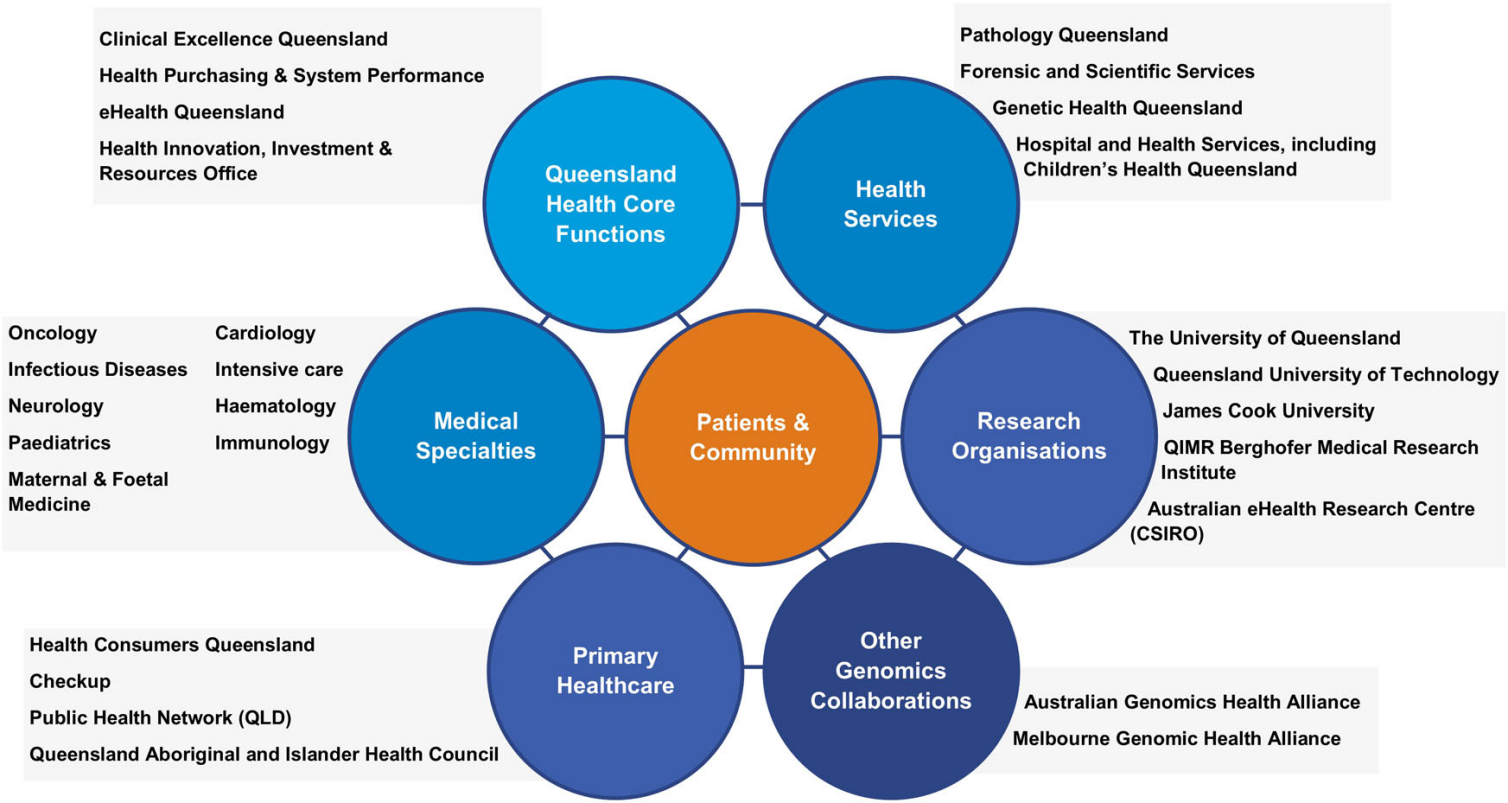
Stakeholders involved with the Queensland Genomics program.

In 2015 the Queensland Government announced a $AU25 million investment to integrate genomics into Queensland’s healthcare system^16^ with the stated objective “to demonstrate the value of genomic medicine in everyday Queensland healthcare”. From this investment, the Queensland Genomics Health Alliance (Queensland Genomics) program was established. Queensland Genomics was a 5-year program (2016–2021) established to bring together the health system, research, academia and consumers (Fig. 1) to accelerate the uptake of genomics through the creation of capability and infrastructure.

Prior to Queensland Genomics, the majority of genomic sequencing was ordered or accessed via the state-wide genetic health service, Genetic Health Queensland, with ad hoc ordering by individual clinicians in other specialties. Testing was largely provided through research projects or ordered directly from private laboratories using department diagnostic budgets. The selection of genomic testing by clinicians who were from non-genetic specialties was generally dependant on the individual’s knowledge of genomic sequencing in their speciality and availability of external services.

### Program design

The structure and management of the Queensland Genomics program were based on an adaptive management philosophy^17^. The adaptive management strategy establishes processes that feedback learnings based on experiences of running the program and have mechanisms to enable change that reflects these learnings. It is applied in complex adaptive systems, such as healthcare^18^, so that programs can operate in situations of uncertainty^19^. This program model was selected as genomics is a discipline experiencing fast technological changes and an evolving knowledge base.

**Fig. 2:**
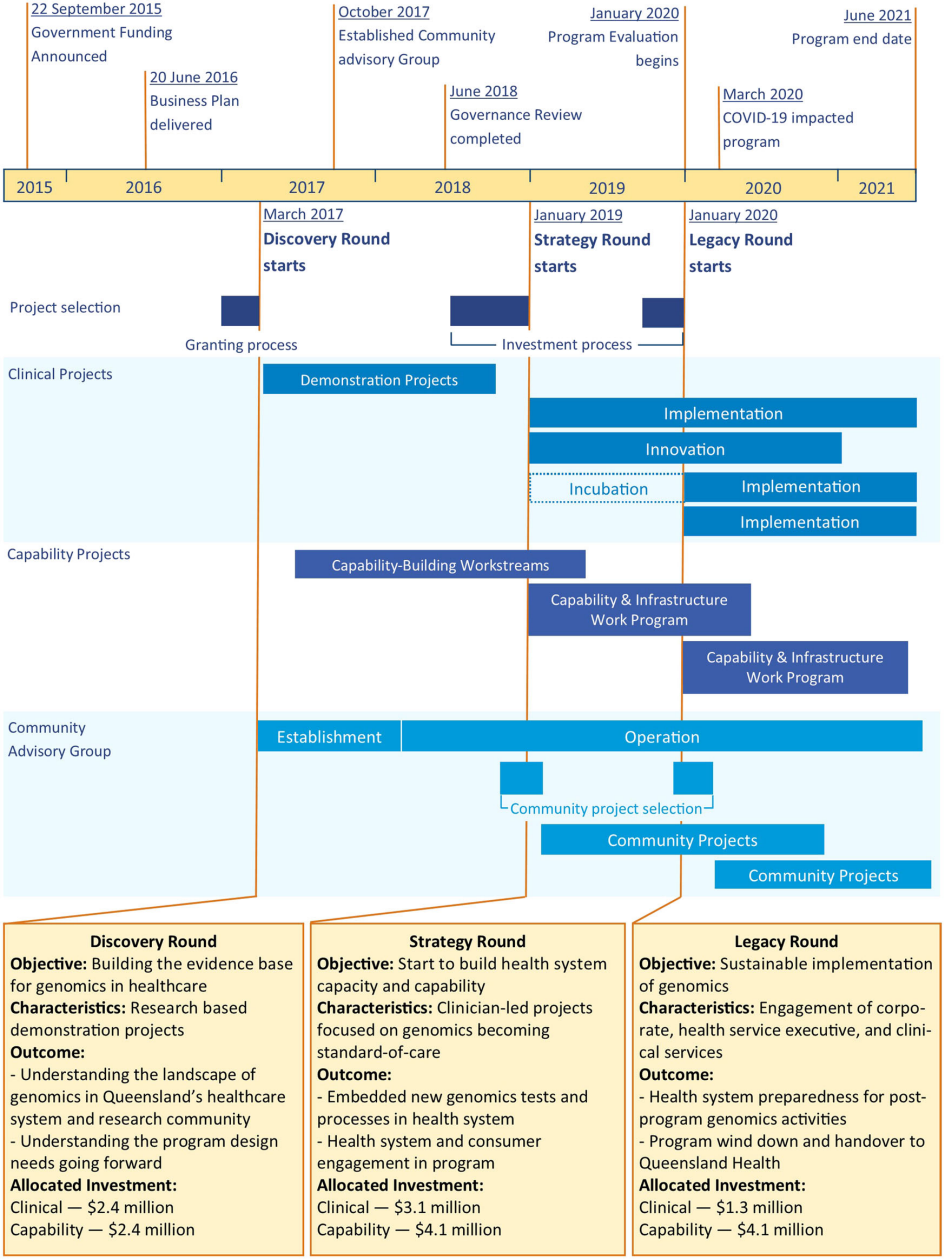
Overview of the Queensland Genomics program structure and timeline. The timing of the key events within Queensland Genomics are shown at the top. The types and timing of the projects are shown in the middle section. The objective, characteristics, outcomes and investment for each round (Discovery, Strategy and Legacy) are in the lower section.

The program contained three sequential rounds called Discovery, Strategy, and Legacy Rounds (Fig. 2). Each round was structured into two arms—Clinical and Capability. The three rounds supported the adaptive management approach, with learnings and needs identified from each Round being used in the selection of projects for the subsequent rounds.

While the structure of the rounds and arms was maintained for the program’s duration, the project selection and expectations changed. The Discovery Round focused on building capacity within the health system through the demonstration of genomics utility by research projects. It was modelled on the structure of other genomic alliances^3,9^. Clinical and Capability projects were selected via an open granting process. The Clinical arm consisted of translational research projects (Table 1). The Capability arm was established to understand needs (present and future) and build capability and capacity for clinical genomics within Queensland’s health system through multi-disciplinary collaborative projects (Table 2).

**Table 1:** Queensland Genomics’ Clinical projects.

	Disease areas	Description	Project type	Recruiting sites	Duration
Demonstration Projects (Discovery Round)	Melanoma	A genomic approach for screening of patients at high risk of melanoma	Demonstration	PAH	2017–2018
	Lung cancer	Bring modern genomics to the management of lung cancer in Queensland	Demonstration	PAH, TSHHS	2017–2018
	Mature onset diabetes in the young (MODY)	Evaluation of targeted genetic testing of MODY in gestational diabetes	Demonstration	Mater	2017–2018
	Hospital-acquired infections	Whole-genome sequencing to track, treat and prevent nosocomial infections	Demonstration	RBWH, PAH	2017–2018
Cancer Portfolio (Strategy & Legacy Rounds)	Myeloid cancer	Improving the survival of children and adults with myeloid cancers	Implementation	RBWH, QCH, SR	2019–2021
	Breast cancer	Q-IMPROvE: Implementation of precision oncology in breast cancer	Innovation	RBWH, Mater, PAH, TSHHS	2020–2021
	Haematology	Comprehensive risk stratification of acute leukaemias by targeted RNA sequencing	Implementation	RBWH, SR	2020–2021
Whole-of-Life Portfolio (Strategy & Legacy Rounds)	Epilepsy	Improving diagnosis and treatment for refractory epilepsy	Implementation	MNHHS, QCH, CHHHS	2019–2021
	Rare diseases	Rapid testing for paediatric intensive care patients	Innovation	QCH, RBWH, SR	2019–2020
	Cardiac genetics	Implementation of cardiac genetics clinics across Queensland	Implementation	TPCH, PAH, CHHHS	2020–2021
	Rare neuro-developmental disorders	Supporting diagnostic access for rare neurodevelopmental and complex multisystem disorders across Queensland	Implementation (via Incubator process)	QCH, MHHS, TSHHS, SR	2020–2021
	Paediatric immunology	Implementation of genomics into an integrated diagnostic and treatment service for primary immune deficiencies and other immune dysregulation syndromes in children	Implementation (via Incubator process)	QCH, SR	2020–2021
Infectious Diseases (Strategy & Legacy Rounds)	Hospital-acquired infections	Whole-genome sequencing to track, treat and prevent nosocomial infections	Implementation	RBWH, PAH	2019–2021
	Sepsis pathogens	Optimising treatment outcomes for children and adults through rapid genome sequencing of sepsis pathogens	Innovation	QCH, RBWH, TPCH, PAH	2019–2021
	Pathogen genomics	Rapid diagnostics that can rule in/out causes of sepsis, rule in/out antibiotic-resistant disease and detect rare or emerging infectious diseases in North Queensland (regional area)	Innovation	TSHHS, CHHHS	2019–2020

*CHHHS* Cairns and Hinterland Hospital and Health Services, *MHHS* Mackay Hospital and Health Services, *MNHHS* Metro North Hospital and Health Services, *PAH* Princes Alexandra Hospital, *QCH* Queensland Children’s Hospital, *RBWH* Royal Brisbane and Women’s Hospital, *SR* Statewide recruitment, *THHS* Townsville Hospital and Health Services, *TPCH* The Prince Charles Hospital.

**Table 2:** Queensland Genomics’ Capability projects and community projects.

	Project name	Description	Duration
Capability-Building Workstream (Discovery Round)	Workforce Development	Development of post-graduate course - Masters in Diagnostic Genomics	2017–2018
	Genomic Testing Innovation	Systems to cover genomics in Queensland Pathology processes including sample collection, processing, analysis, tracking, and reporting	2017–2018
	Genomic Information Management	Architecture for Queensland’s genomic information management, and the standards, policies and procedures to support a common infrastructure for the management and use of genomic data	2017–2018
	Evaluation of Clinical Genomics	Assess the health economics of incorporating genomics into the health system based on Discovery Round projects	2017–2018
	Ethical Legal and Social Implications of Genomics (ELSI)	Develop a series of guidelines, policies and advice to support; community engagement, consent, research, justice and clinical use of genomics, including GenetiQs	2017–2018
Capability & Infrastructure Work Program (Strategy & Legacy Rounds)	Primary Care and General Practise in Genomics Eeducation	Online training modules on genomics in primary care to upskill Queensland’s general practitioner workforce in genomic medicine, to prepare for the state-wide integration of genomic treatment in primary care	2019–2021
	Nursing and Midwifery Genomics Education and Knowledge	Development of education and scope of practice frameworks and resources for integrating genomics into nursing and midwifery practice in Queensland	2019–2021
	Variant Curation Workshops	Deliver a national cancer variant curation workshop for clinicians working in cancer genomics; and a hereditary variant curation workshop to build core capabilities in Queensland	2019
	Pathology Queensland Centre for Integrated Genomics	Building and expand Pathology Queensland’s capabilities in genomics sequencing services capacity and genomics-based diagnostic activities	2019–2021
	Genomic Institute	Undertaking planning for the establishment of a Statewide Genomic Institute to develop integration across clinical services, education and training, research and innovation, data and technology, academic, and industry partnerships	2019–2021
	Integrating Genomics into the ieMR	Partner with Clinical Excellence Queensland and eHealth Queensland to ensure Queensland’s integrated electronic Medical Record (ieMR) supports genetic and genomic medicine requirements	2019
	Longitudinal Information Management and Advanced Decision Support	Strategic approach to managing genomic information captured from clinical diagnostics, and exploring the application of artificial intelligence in medical genomics	2019
	Evaluation of Clinical Genomics	Assessing the health economics of incorporating genomics into the health system based on Strategy Round Projects.	2019–2021
	Indigenous Genomics Health Literacy (IG-HeLP)	In partnership with Aboriginal and Torres Strait Islander peoples develop culturally appropriate genomics communication materials	2019–2020
	Statewide Consent and Ethics for Genomics	Establish standardised approaches to consent for clinical genomics in Queensland	2019
	Queensland Online Oncology Tool (QOOL) enhancement - Epilepsy Genomics Project	Development of the QOOL application to capture clinical data to inform clinical decision making in Epilepsy	2019–2020
	Queensland Online Oncology tool (QOOL)	Upgrade to the existing QOOL application to incorporate genomic clinical information to facilitate multi-disciplinary team meetings (MDT)	2020–2021
	Queensland Genomics Program Evaluation	External evaluation of genomics in Queensland to support decision making for future investments into clinical genomics	2020–2021
	Aboriginal and Torres Strait Islander Referral Pathways	Design a coordinated care model for Aboriginal and Torres Strait Islander peoples to improve referral pathways to Genetic Health Queensland	2020–2021
	Children and Genomics Strategy	Support Children’s Health Queensland to develop a strategy for the ongoing adoption of genomics	2020–2021
	Children’s Hospital Queensland Cancer Genomics Service	Support the development of Children’s Cancer Genomics Services via the QCH Cancer Department with a focus on family-led services in a “plan and do” approach e.g. sequence whilst planning	2020–2021
Community Advisory Group Projects (Strategy & Legacy Rounds)	Patient Communication Toolkit	Develop resources for patients to support their ability to access and engage with Queensland’s genetic health services	2019–2020
	Queensland Patient’s Journeys in Genomics	Engaging with patients to understand their experiences with accessing genetic health services in Queensland	2019–2020
	Health Consumers Queensland Forum & Evaluation	Evaluation of consumer knowledge of genomics at Health Consumers Queensland forum	2019–2020
	Genomic Literacy in Multicultural (GLiM) Queensland	Provide education to multicultural health workers in genetic health services in Queensland; via training for Bilingual Community Health Workers and Medical Interpreters	2019–2021
	Medical Interpreter Training Evaluation	Research project evaluating the outcomes of GLiM’s medical interpreter training sessions in 2019 & 2020	2020–2021
	Queensland Genetic Support Network scoping	Engage with patient stakeholders across Queensland to determine their needs for a genetic support network.	2020–2021
	Queensland Genomics Community Advisory Group Publication	Develop and submit a journal article for peer-review publication presenting the structure and activities of the Queensland Genomics Community Advisory Group	2021

The Strategy and Legacy Rounds were established after the governance review and focused on patient outcomes through sustainable clinical genomics implementation, clinical innovation to match future needs, and targeted health system capability building. Selection of investments changed from a granting process to a multi-stage, co-design process with the project teams and involved stakeholders, health services and Clinical Networks. Within the Strategy and Legacy Rounds the clinical projects were categorised as implementation, innovation or incubation projects. Implementation and incubation projects applied the best evidence to clinical practice or developed evidence in clinical practise, respectively, with the aim to become standard-of-care following completion of the program. Incubation projects were those not ready for investment due to gaps identified within the co-design process. However, with support, they had the potential to be ready for funding later during the Legacy Round.

The Capability arm of the program was restructured to better support Queensland Health’s state-wide services and core business functions to build their ability to undertake and utilise genomics, as well as continuing to support the needs of Clinical projects. In both the Strategy and Legacy Rounds, the projects moved from five large collaborative projects to smaller commissioned work to deliver specific projects with health service-focused objectives (Table 2).

### Governance review and program restructure

The Queensland Genomics Business Plan specified a review of governance, structure and operations at the 18-month time point. The governance review gathered information by interviewing key stakeholders from; Hospital and Health Services, clinicians and clinical services, Queensland Health executive branches, consumers, academia and research institutes. The interviews focused on what was needed for the program to support the continual adoption of genomics into Queensland’s healthcare system by exploring; executive and clinical network engagement, evidence, investment outcomes, health system readiness and requirements, and options for private-public partnerships.

The governance review identified several key findings; (1) if the original program structure was continued, it was unlikely to affect sustained uptake of genomics into the health system once the program ended, (2) the first round of projects had insufficient engagement with the health system as the program was geared heavily towards research rather than healthcare transformation, and (3) the original plan modelled Queensland Genomics to other genomics alliances, but this assumed that these programs operated in the same context and with the same funding structure as Queensland. Therefore continuing this model would not meet the needs of the Queensland health system.

In response, the program was restructured prior to the start of the Strategy Round to have an emphasis on health implementation with a focus on Queensland Health ownership of outcomes, and sponsorship of projects and initiatives. The updated aim of the program became to “accelerate the translation of genomics into clinical practice”. The restructure included changes to: governance structure and responsibilities; project selection processes; Queensland Genomics involvement in the management of projects and strategic interactions with Queensland Health; and requirements for projects to engage with health services. This new process was designed by the Queensland Genomics business team in consultation with Queensland Health Executive leadership and approved by the Governance Oversight Group.

The restructure resulted in Queensland Genomics moving from its initial research, demonstration and ‘discovery’ orientation to a focus on; (1) sustainable clinical genomics implementation and incubation, (2) clinical innovation with potential to match future needs, and (3) targeted health system capability building. This is reflected in the main outcomes of the program (Table 3).

**Table 3:** High-level impacts of Queensland Genomics on genomic implementation in Queensland’s health system.

Impact	Description
Ownership	− Handover of genomics activities and output to relevant departments for continuedstewardship. − Genomics in Queensland Health Strategy for post-program implementation of genomics in the health system at multiple administrative levels.
Access to genomic testing	−Developed pathways for accessing genomics in Cancer, Paediatrics, Cardiology, and Infection and Infection Prevention and Control.
Education	− Workforce development in genomics in the areas of; nursing, midwifery, variant curation, infection prevention and control, medical interpreters, bilingual community health workers, and general practitioners.
Building capacity	− Next generation sequencing infrastructure. National accreditation of state-wide pathology service for clinical use of whole-exome sequencing and whole-genome sequencing. − Development of Pathology Queensland Strategic plan.

### Management systems and program governance

**Fig. 3:**
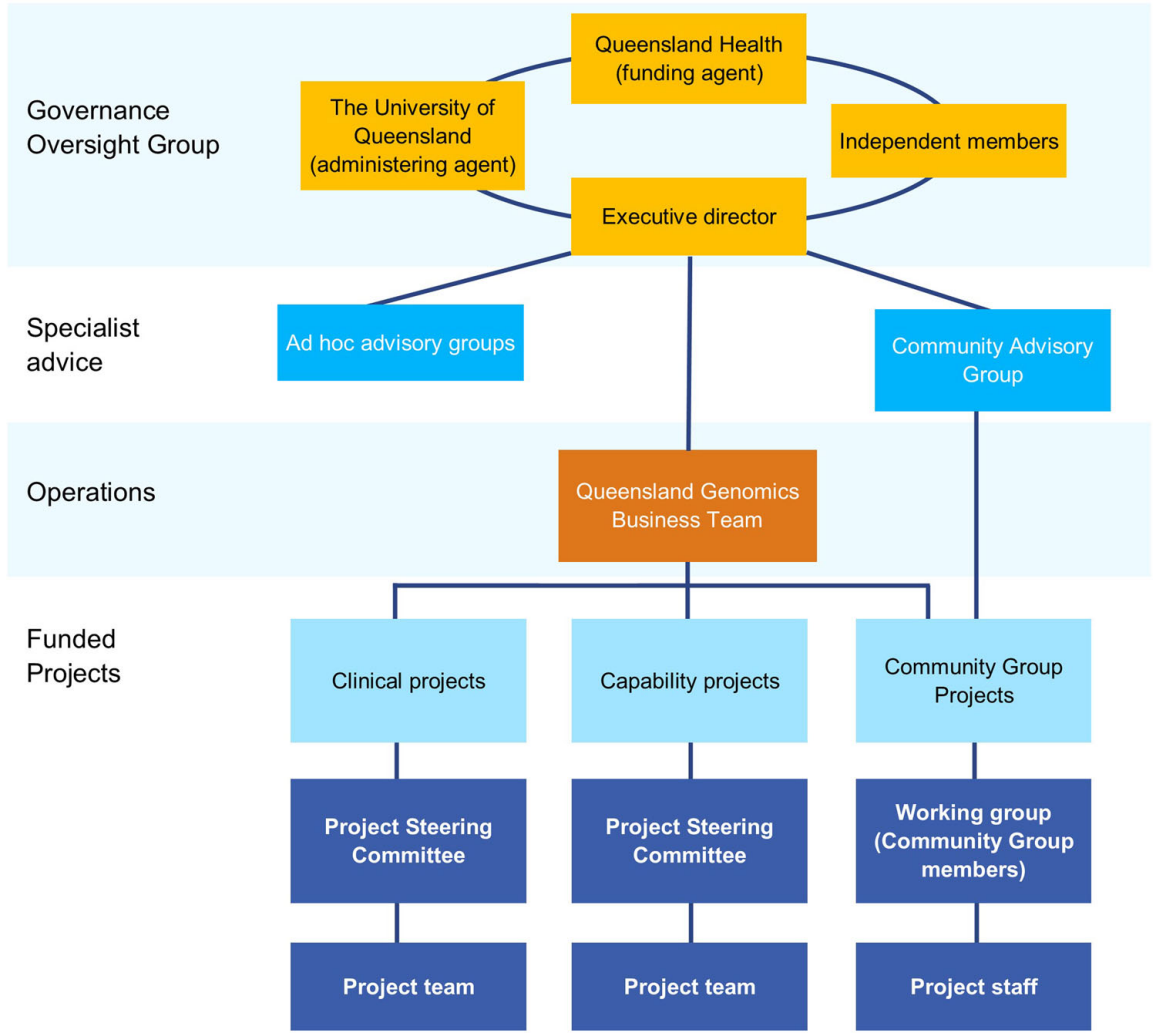
Queensland Genomics post-review governance structure. The program was overseen by a Governance Oversight Group, with a business team managing design and delivery, contract and administration and communication of the funded projects.

Queensland Genomics was established as an external entity to the health system that was managed via a university (support for finance, human resources, and operational space). This enabled the program’s operations to function independently with a governance oversight group that reported to Queensland’s Department of Health. The main roles of the Governance Oversight Group were approving Queensland Genomics processes and endorsing decisions. The Queensland Genomics business team were not genomics experts or clinicians and were recruited to provide leadership in health transformation, business and technology strategy, communication and engagement, and innovation. They supported the individual projects through project design and delivery, contract administration, and establishing connections between projects and Queensland Health. As needed, specialist expertise was drawn upon using ad hoc advisory panels and the program’s community advisory group (Fig. 3).

Following the governance review, Clinical projects were led by a practising clinician and, where possible, Clinical and Capability projects were sponsored and led by Queensland Health or Hospital and Health Services (Tables 1 and 2). Each project reported to and gained advice and direction from a Project Steering Committee (PSC). Typically, a PSC included a mix of project team members, Queensland Health representatives, community advocates, external technical experts, and/or Queensland Genomics business team members. This individual project oversight aimed to engage Queensland Health in the process to ensure (1) project objectives fit the end-user’s needs, and (2) appropriate internal stakeholders were engaged to prompt post-program legacy for the project outcomes.

### Community advisory group

The Queensland Genomics program had an overarching emphasis that projects should be patient-orientated. The Queensland Genomics Community Advisory Group (CAG) was established at the start of the program (Fig. 2) and consisted of up to 11 members representing patients, carers, consumer advocates, clinicians and researchers. It was a requirement for Clinical and Capability projects to engage with the patient community in project development and/or implementation. The CAG provided input for projects if they had no existing patient community organisations to engage with. In the Strategy and Legacy Rounds, the CAG received funding from Queensland Genomics to identify, develop, and deliver its own community-led projects (Table 2).

## Program activities

In the Discovery Round, four Clinical project grants were awarded in areas of cancer, common chronic diseases, and infectious diseases (Table 1). Five Capability projects were funded (Table 2). The Capability projects generally containing a series of interrelated sub-projects and multiple partner collaboration^20–22^.

In the Strategy and Legacy Rounds, 11 Clinical projects were funded. These projects were organised into three clinical portfolio areas; Cancer, Whole-of-Life, and Infectious Diseases (Table 1)^23–26^. Structuring projects into portfolios enabled sharing of resources, processes and learning, enabling sustainable implementation. Consequently, the individual projects contributed to broader portfolio-specific objectives around: implementation of a state-wide workforce model for genomics; establishment of a genomics clinical advisory service and a molecular tumour board for cancer; proposed data integration solution for the integrated electronic Medical Record (ieMR); and providing evidence for genomics as a routine tool in infectious disease outbreaks.

These portfolio-specific objectives came from commonalities identified in Clinical projects’ plans, or needs identified by capabilities projects, health services or the Queensland Genomics business team. These objectives fostered collaboration by building and funding co-dependencies between projects and by association with their clinical services. Thereby, lifting capability, creating value for each partner, and creating inter-departmental connections. This approach did require additional time and effort to develop collaborations and, in some instances, was met with reluctance from project members. Implementation of this approach required additional resourcing at the program’s administrative level, plus support and funding for the project partners.

Sixteen Capability projects were commissioned within the Strategy and Legacy Rounds (Table 2). Projects focused on (1) preparation of the health system for genomics, or (2) support or evaluation of Clinical projects^27–35^. In addition, seven projects designed by the CAG were funded in the Strategy and Legacy Rounds (Table 2) that focused on areas of need for the patient community and patient support mechanisms within the health system^36^.

## Lessons learned from an adaptive approach

The aim, and ultimate achievement, of Queensland Genomics was the development of capacity, infrastructure and policies to enable safe, appropriate and consistent application of genomics in Queensland’s public health system. The choice to design the Queensland Genomics program using an adaptive management approach had a number of benefits particularly to the program’s flexibility and adaptability. However, this direction was not without its challenges. The lesson, and actions suggested, based on Queensland Genomics’ experiences are summarised in Table 4. These observations centred around; how stakeholders were engaged and supported to contribute, mechanisms of the program and project design that enabled flexibility, and support provided to enable the timely establishment of expectations.

**Table 4:** Lessons from the Queensland Genomics program.

Category	Description	Program experience	Suggested actions
People	Networking opportunities within program	Provide opportunities for inter-project networking, especially between funding rounds and geographically distance project teams.	− All of program meetings or symposiums − Facilitate meeting between projects with similar needs or intersecting interests − Seminars series
	Learning opportunties	Genomics is complex and new to many stakeholders. Queensland Genomics used the experienced stakeholder to upskill others.	− Multi-disciplinary team meetings to discuss project cases that are open to non-project members − Supporting projects to develop education resources for their discipline
Project implementation	Project coordinators	Projects struggled to establish themselves without a project coordinator, and this role was not factored into budgets or hiring was delayed.	− Include project co-ordinators as a line item in budget or as an expectation in project development or granting documentation﻿ − Support sharing of project co-ordinators as a resource across multiple projects
	Project specific-milestones	The main milestone for clinical projects was the number of patients sequenced, however this was not appropriate for some. For health implementation the process of establishing a health system embedded process is critically important.	− Plan projects milestones or deliverables to focus on areas that impactful for the individual project
Program design	Consumer and Community Engagement	Patient and health consumer input was beneficial for identifying unmet needs within the program and maintaining a patient-centred focus to the body of work.	− Establish a program wide consumer and community engagement plan with mechanism and expectations for project implementation − Include community and consumer engagement as a line item in budget − Supporting community-led, designed or initiated projects
	Flexibility in program design	Adaptive approach allowed the program to grow and develop the program as knowledge of genomics in the health system increased.	− Mechanisms for approval of new initiatives or changes to program by Governance Oversight Group − Pathways for projects to suggest new ideas or initiatives
	Program support during project development	More specialities can be involved in health system implementation program if there is are opportunity for supporting project development.	− Program engagement with Executive, clinical specialties and clinical networks that have not previously shown interest in genomics − Incubator process to support under-developed projects to a stage where they can be funded
	Program wide data sharing	Plan internal and external sharing of data early in the program.	− Contractual requirement for incorporating data sharing − Standardised program template documents for data sharing

## Benefits

### Time to determine program direction

The Discovery Round was designed as a time to capture lessons from program activities, and utilise this information to assess and make changes to the program direction. Critically, the governance review found that the program was unlikely to affect sustained uptake of genomics into Queensland’s health system once the program ended if it continued along a research orientated path. This led to a program-wide restructuring whereby the program envisioned a future where genomics was business-as-usual in Queensland’s health system. This led to a program of work in the Strategy and Legacy Rounds that was customised to the health system and its specific health implementation needs, with ongoing smaller adjustments as new opportunities were identified. The recognition that the program’s initial direction would not facilitate health system implementation and the capacity to pivot the program in response to the recommendation was only possible due to the original program design. The design provided time and assessment mechanisms to determine the most appropriate direction for the program.

### Establish health system ownership

Health system ownership of program activities was imperative to ensure post-program uptake of genomics. For successful implementation within a health system upskilling and supporting providers needed to occur across multiple interrelated services. For example, Clinical projects were run by medical specialities who at the completion of the project may be responsible for test ordering and management of patients, but sample processing and sequencing are the responsibility of pathology services, and clinical data management is the responsibility of information systems. The adaptive approach allowed for health services’ needs in the uptake of genomics to be identified whilst projects were running, and project plans to be revised accordingly through engagement with those future owners.

### Multiple opportunities for increased stakeholder buy-in

Genomics is a discipline that requires a level of genetic literacy before people can contribute to discussions^2^. It is an inherent issue in the adoption of new technologies in complex healthcare systems that stakeholders without pre-existing knowledge or involvement are difficult to effectively engage. The Queensland Genomics program was initiated with stakeholder consultation with predominantly researchers, and clinical specialists, services and administrators that already had involvement with clinical genetics or genomics. However, it has been shown that high-level stakeholders, such as healthcare executives and regulators, need to be engaged for long-term adoption of genomics in clinical care^37^. The use of an adaptive model allowed the program to be established with the support and input of engaged stakeholders, whilst having the ability to engage and upskill new stakeholders during the program.

### Responsive to external influences and events

The adaptive design allowed the Capability and Clinical arms to reflect and respond to the changing landscape of clinical genomics in Australia. During the program’s lifecycle the Australian clinical genome sequencing market fundamentally changed. This demonstrated a national vulnerability in accredited sequencing capacity and affected Queensland Health’s plans for clinical genomics. There was also the deployment of a federal program to standardise aspects of clinical genomics across states in response to the National Health Genomics Policy Framework^38^. The adaptive strategy enabled Queensland Genomics to accommodate these changes into the program and post-program planning by providing investment, data management processes and workforce planning around genomic sequencing into Queensland Health’s statewide public pathology service.

## Challenges

### Change management

Pivoting the program to reflect the governance review findings required substantial change management for both funded stakeholders and program administrators. The program was established with a granting and research style funding and reporting structure that was familiar to the Discovery Round stakeholders that had pre-existing experience with these processes. When the governance review identified limitations in the program’s ability to enact health system implementation, the program structure was changed from a granting process to a commissioning process with focus on Queensland Health as the beneficiary. For existing external stakeholders the change in funding structure and program expectations was difficult to manage. There was also a significant change in responsibilities and work load for the program administration, as they shifted from a largely oversight and administration role to being active in project deployment, system capability development, and coordinating a genomics ecosystem for Queensland.

### External structures and processes

A challenge with adaptive management is that it can impose unfamiliar demands on the managing organisation^39^. Much of this was avoided by implementing the program outside the health system bureaucracy to enable the development of an agile program business structure. However, the program struggled to maintain this agile business structure whilst working within standard government processes of the health system. This influenced project approvals, contracting, intellectual property agreements, ethical and governance approvals, and ultimately, the timeline for implementation of the program. This was exacerbated by the post-governance review changes.

### Competing with health system priorities

Engaging organisation leadership is critical for the successful uptake of genomics in clinical settings^37,40^. Yet, as genomics isn’t mainstream it was difficult to convince executive leaders, with health system-wide responsibilities, that genomics is worthy of their time and attention. The Queensland Genomics program made numerous attempts throughout the program to engage executive leaders in program activities and post-program planning for clinical genomics, with varying degrees of success. In the case of post-program planning the impending program end date created an urgency that motivated action within Queensland Health, in the form of an Executive-level working group to develop a State policy position for genomics in Queensland as a precursor for the development of funding frameworks.

## Conclusions

It is recognised that genomics as a clinical test requires system-wide change to be effectively implemented into a health system^8^. By structuring Queensland Genomics using an adaptive management philosophy, the program had the opportunity to collect and apply lessons from earlier activities and feed this information into an engagement strategy that could affect real change to the health system from within the program. This ultimately enabled the program to evolve over time and create a program that was relevant to Queensland Health’s system needs. Overall, this approach:

- Prompted broad health system awareness, knowledge and readiness for clinical genomics - not only at clinical level, but also at an operational and executive level
- Coordinated involvement from health information management, infrastructure planning, information technology, pathology services, patient services, multiple medical disciplines, and health policy
- Focused on developing a program that can deliver post-program sustainability within existing Queensland Health governance and funding frameworks
- Planned for post-program continuation of genomics within clinical services and health systems.

Through adapting funding structure and program development to enable a customised approach to genomics implementation needs, Queensland Genomics has prompted administration of funding and resources that reflected health system stakeholder needs and system readiness.
